# Influence of posterior corneal astigmatism on postoperative refractive astigmatism in pseudophakic eyes after cataract surgery

**DOI:** 10.1186/s12886-016-0391-1

**Published:** 2016-12-01

**Authors:** Maki Sano, Takahiro Hiraoka, Yuta Ueno, Hideo Itagaki, Tomohiro Ogami, Tetsuro Oshika

**Affiliations:** 1 Department of Ophthalmology, University of Tsukuba Hospital, 2-1-1 Amakubo, Tsukuba, Ibaraki 305-8576 Japan; 2 Hitachi General Hospital, 2-1-1 Jonancho, Hitachi, Ibaraki 317-0077 Japan; 3 Seinan Medical Center hospital, 2190 Sakaimachi, Sashimagun, Ibaraki 306-0433 Japan; 4 Department of Ophthalmology, Faculty of Medicine, University of Tsukuba, 2-1-1 Amakubo, Tsukuba, Ibaraki 305-8576 Japan

**Keywords:** Posterior astigmatism, Cataract surgery, Toric intraocular lens

## Abstract

**Background:**

To examine the influence of posterior corneal astigmatism on postoperative refractive astigmatism in pseudophakic eyes after cataract surgery.

**Methods:**

The study enrolled 64 pseudophakic eyes of 50 patients (71.8 ± 9.9 years old, mean ± standard deviation) who had undergone phacoemulsification with non-toric IOL implantation. Refractive astigmatism was measured using an auto ref-keratometer with a 0.01- diopter (D) scale. Two types of corneal astigmatism were calculated using anterior segment optical coherence tomography; keratometric and total corneal astigmatism. Keratometric astigmatism was obtained based on anterior corneal curvature alone and total corneal astigmatism was calculated using both anterior and posterior corneal curvatures. The difference between refractive and corneal astigmatism was computed as the vector difference using 1) refractive and keratometric astigmatism and 2) refractive and total corneal astigmatism.

**Results:**

The mean refractive, keratometric, and total corneal astigmatism was 0.92 ± 0.48 D, 0.87 ± 0.44 D, and 0.94 ± 0.46 D, respectively. The difference between refractive and keratometric astigmatism (0.70 ± 0.40 D, mean vector of 0.30 D axis 164°) was significantly larger than the difference between refractive and total corneal astigmatism (0.63 ± 0.38 D, mean vector of 0.12 D axis 137°) (*P* = .019).

**Conclusions:**

The difference between refractive and total corneal astigmatism, calculated using both anterior and posterior corneal curvatures, was significantly smaller than the difference between refractive and keratometric astigmatism using anterior corneal astigmatism alone, implying that the latter overestimates the true postoperative refractive astigmatism and can cause cylindrical inaccuracy after cataract surgery.

## Background

It has been reported that uncorrected astigmatism of greater than one diopter (D) in magnitude significantly deteriorates uncorrected visual acuity in pseudophakic eyes [1]. The introduction of toric intraocular lens (IOL) technology has made it possible to offer better and more stable uncorrected visual acuity to patients with astigmatism. In clinical practice, however, some patients still present with postoperative refractive astigmatism of unknown origin even with toric IOL implantation, and the accuracy of preoperative measurements of corneal astigmatism is often discussed [2, 3].

Both anterior and posterior corneal curvatures contribute to total corneal astigmatism [4], but less attention has been directed to posterior corneal curvature [4–10]. This is because traditionally anterior and posterior corneal surfaces in normal eyes were thought to be almost parallel in shape. In addition, refractive power of the posterior surface is much smaller than the anterior surface due to the small difference in refractive index between the corneal stroma and the aqueous humor. Thus, in general, ophthalmologists tended to believe that detailed examination of posterior corneal shape and curve is not necessary [7]. Therefore, keratometric astigmatism which is calculated based on anterior corneal measurements only has been used clinically to represent total corneal astigmatism, assuming a fixed posterior/anterior curvature ratio to estimate the contribution of posterior corneal power [4].

Newer technologies, such as slit-scanning videokeratoscope, Scheimpflug device, and anterior segment optical coherence tomography (AS-OCT), are now available for measuring anterior as well as posterior corneal shapes [4]. Results obtained with these devices demonstrated that keratometric astigmatism calculated based on the measurements of anterior corneal surface alone significantly differ from that based on both anterior and posterior corneal measurements [4, 5]. The posterior corneal surface tends toward against-the-rule astigmatism pattern in comparison with the anterior corneal surface [7]. Therefore, in eyes with with-the-rule astigmatism, keratometric astigmatism overestimates total corneal astigmatism, whereas in eyes with against-the-rule astigmatism, keratometric astigmatism underestimates total corneal astigmatism [4, 7, 10]. These discrepancies seem to be explained by the fact that corneal thickness profile is not uniform between horizontal and vertical directions, i.e., the cornea is thicker in the vertical than the horizontal directions [11]. Thus, it is not always true that the anterior and posterior corneal curvatures have a constant and linear relationship [9, 11], and posterior corneal astigmatism should be at least partially responsible for postoperative refractive astigmatism of unknown origin [2, 3].

The above findings highlight the need to clarify the effect of posterior corneal astigmatism on postoperative refractive astigmatism after cataract surgery. Several previous reports have already demonstrated the relationship between posterior corneal astigmatism and total corneal astigmatism [4, 5, 12]. However, the relationship between posterior corneal astigmatism and postoperative refractive astigmatism of unknown origin in patients undergoing cataract surgery has not been examined in detail. In this study, we investigated the influence of posterior corneal astigmatism on postoperative refractive astigmatism in pseudophakic eyes with non-toric IOL implantation.

## Methods

This study included consecutive eligible patients who had undergone phacoemulsification with non-toric IOL implantation at the University of Tsukuba Hospital from November 2012 to March 2013. Patients who had corneal or retinal disease and a history of ocular surgery other than cataract surgery or ocular injury were excluded. Patients were also excluded if they met any of the following criteria: postoperative decimal best-corrected visual acuity of less than 0.8 (decimal) (20/25 snellen), history of toric IOL implantation, or cases with surgical complication. Informed consent was obtained from all patients. The research was approved by the Institutional Review Board of the University of Tsukuba Hospital and conducted according to the tenets of the Declaration of Helsinki.

The preoperative evaluation included measurements of objective refractive power obtained using an auto ref-keratometer (RC-5000, Tomey Corporation, Nagoya, Japan) with a 0.01-D scale, axial length measured by contact applanation ultrasound (AL-1100, Tomey Corporation, Nagoya, Japan), and keratometric and total corneal powers measured by an AS-OCT (SS-1000, CASIA, Tomey Corporation, Nagoya, Japan). We took automatically-calculated values of total corneal power from the AS-OCT based on the actual measures of anterior and posterior corneal power.

A standard phacoemulsification technique was performed through a 3-mm superior sclerocorneal one-plane incision under topical anesthesia. Aspheric and non-toric IOLs were implanted in all patients.

One month after surgery, we measured postoperative refractive power using the RC-5000 with a 0.01-D scale and keratometric power and total corneal powers using the AS-OCT, and then assessed the correlation among postoperative refractive, keratometric, and total corneal astigmatism. In addition, the vector differences between postoperative refractive astigmatism and two types of corneal astigmatism (keratometric and total corneal astigmatism) were calculated and compared with each other. In this study, all measurements were based on the data from the annular ring with 3 mm in diameter around the corneal apex.

### AS-OCT

The AS-OCT is a non-contact, non-invasive three-dimensional imaging system based on the principle of “Swept Source” OCT. This system uses light of 1,310-nm wavelength and achieves resolutions of 10 μm (axial) and 30 μm (transverse) to obtain 30,000 axial-scans per second. The scan range diameter is 10.0 mm, and 16 radial cross-sectional images were obtained within 0.34 s per measurement, with each image containing 512 measurement points [13–15]. All measurements were taken by experienced examiners (MS and YU). Two images were obtained for each eye, and the better image was selected for data analysis.

### Corneal astigmatism calculation

Keratometric power was calculated using the keratometric index (1.3375) and the radius of anterior corneal curvature, while total corneal power was calculated based on the refractive power of the anterior and posterior corneal surface as well as corneal thickness.

$$ \begin{array}{l}\mathrm{keratometric}\ \mathrm{power} = \left(1.3375\ \hbox{-}\ 1.0\right)/\mathrm{r}\\ {}\kern2em \mathrm{keratometric}\ \mathrm{index} = 1.3375\\ {}\kern2em \mathrm{r} = \mathrm{r}\mathrm{adius}\ \mathrm{of}\ \mathrm{anterior}\ \mathrm{corneal}\ \mathrm{curvature}\end{array} $$

$$ \begin{array}{l}\mathrm{total}\ \mathrm{cornea}\mathrm{l}\ \mathrm{power} = \mathrm{P}\mathrm{a} + \mathrm{P}\mathrm{b} - \mathrm{d}\times \mathrm{P}\mathrm{a}\times \mathrm{P}\mathrm{b}/1.376\\ {}\kern2em \mathrm{P}\mathrm{a} = \mathrm{refractive}\ \mathrm{power}\ \mathrm{of}\ \mathrm{the}\ \mathrm{a}\mathrm{nterior}\ \mathrm{cornea}\\ {}\kern2em \mathrm{P}\mathrm{b} = \mathrm{refractive}\ \mathrm{power}\ \mathrm{of}\ \mathrm{the}\ \mathrm{posterior}\ \mathrm{cornea}\\ {}\kern2em \mathrm{d} = \mathrm{cornea}\mathrm{l}\ \mathrm{thickness}\\ {}\kern2em \mathrm{refractive}\ \mathrm{index}\ \mathrm{of}\ \mathrm{the}\ \mathrm{cornea} = 1.376\end{array} $$

In this study, the AS-OCT was used to measure and calculate keratometric and total corneal astigmatism. Keratometric astigmatism was calculated as the difference in keratometric power between the steepest and flattest meridians, whereas total corneal astigmatism was calculated based on total corneal power without regard to keratometric power.

### Vector difference between refractive and corneal astigmatism

In this study, we computed vector difference between postoperative refractive astigmatism and each of two types of corneal astigmatism (keratometric and total corneal astigmatism) using equations below [16], and these differences were compared with each other to simulate the influence of actual posterior corneal astigmatism on refractive astigmatism in pseudophakic eyes after non-toric IOL implantation, in which internal astigmatism induced by IOL itself is theoretically regarded as 0 D.

$$ {\mathrm{x}}_{\mathrm{r}}=\mathrm{refractive}\ \mathrm{astigmatism} \ast \mathrm{C}\mathrm{o}\mathrm{s}\ \left(2\ast \mathrm{axis}\right) $$

$$ {\mathrm{y}}_{\mathrm{r}}=\mathrm{refractive}\ \mathrm{astigmatism}\ast \mathrm{Sin}\ \left(2\ast \mathrm{axis}\right) $$

$$ {\mathrm{x}}_{\mathrm{c}}=\mathrm{corneal}\ \mathrm{astigmatism}\ast \mathrm{C}\mathrm{o}\mathrm{s}\ \left(2\ast \mathrm{axis}\right) $$

$$ {\mathrm{y}}_{\mathrm{c}} = \mathrm{corneal}\ \mathrm{astigmatism}\ast \mathrm{Sin}\ \left(2\ast \mathrm{axis}\right) $$

In the formulas, the angle of the axis of astigmatism is doubled to give the correct x and y values.

$$ \mathrm{Cylinder} = \sqrt{\left(\mathrm{x}\mathrm{r}\ \hbox{-}\ \mathrm{x}\mathrm{c}\right)2 + \left(\mathrm{yr}\ \hbox{-}\ \mathrm{y}\mathrm{c}\right)2} $$

$$ \mathrm{Angle} = 1/2\ast \mathrm{Arc}\  \tan\ \left(\mathrm{y}/\mathrm{x}\right) $$

$$ \begin{array}{l}\mathrm{If}\ \mathrm{x}\ \mathrm{and}\ \mathrm{y} > 0\kern2.49em \mathrm{then}\ \mathrm{Axis} = \mathrm{Angle}\\ {}\mathrm{If}\ \mathrm{x} < 0\kern4.73em \mathrm{then}\ \mathrm{Axis} = \mathrm{Angle} + 90{}^{\circ}\\ {}\mathrm{If}\ \mathrm{x} > 0\ \mathrm{and}\ \mathrm{y} < 0\kern1.12em \mathrm{then}\ \mathrm{Axis} = \mathrm{Angle} + 180{}^{\circ}\end{array} $$

Refractive astigmatism was corrected to the corneal plane using the following equation: Fc = (1000 x Fs)/{1000 - (Fs x d)} (Fc = refractive power (D) at the corneal plane, Fs = refractive power (D) at the spectacle plane, and d = vertex distance (12 mm)) [17].

### Statistical analyses

Two types of corneal astigmatism (keratometric and total corneal astigmatism) were compared using the paired *t*-test. The mean magnitude of difference between refractive and keratometric astigmatism, and difference between refractive and total corneal astigmatism was also compared using the paired *t*-test. The mean differences in magnitude between refractive and each of two types of corneal astigmatism according to the types of preoperative keratometric astigmatism, such as ATR, WTR or oblique astigmatism were compared using the Wilcoxon signed-rank test. Using Pearson’s correlation and Bland-Altman plots, the correlation between postoperative refractive and two types of corneal astigmatism were examined. In addition, after all patients were divided into two groups based on the magnitude of difference between postoperative refractive and total corneal astigmatism, various parameters such as age, spherical equivalent refraction, refractive astigmatism, keratometric astigmatism, total corneal astigmatism, axial length, and IOL power were compared between the two groups with greater than 0.5 D and less than 0.5 D difference using Student’s *t*-test. *P* values < 0.05 were considered statistically significant. Statistical analyses were carried out using StatView version 5.0 (SAS Inc., Cary, NC).

## Results

**Fig. 1:**
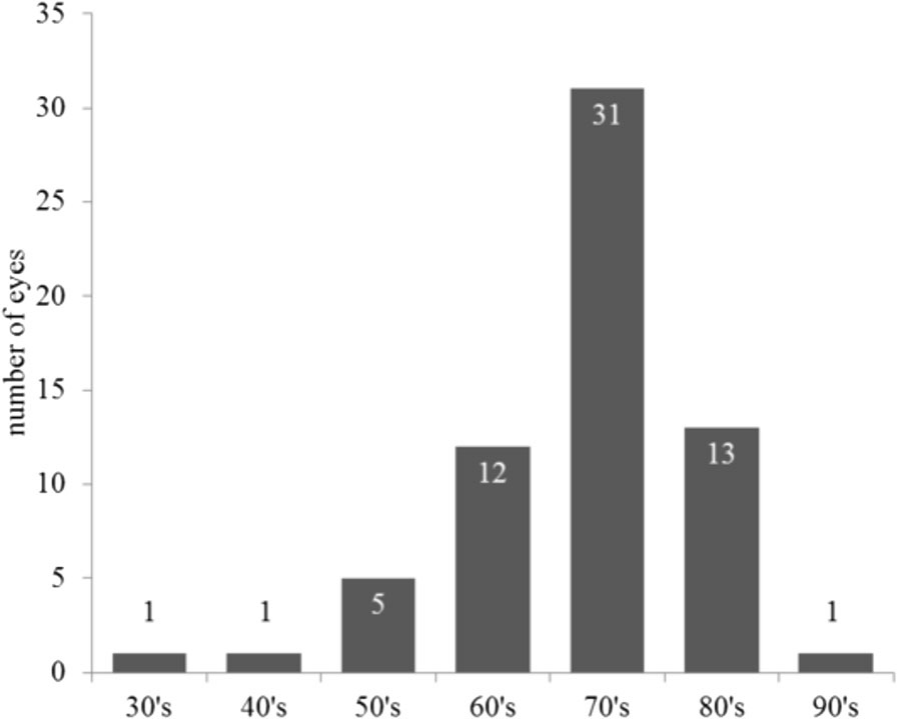
The age distribution of patients was shown

This study enrolled 64 eyes (33 right; 51.6%) of 50 patients (25 women; 50.0%) with a mean age of 71.8 ± 9.9 (SD: standard deviation) years (range 33 to 92 years). The age distribution of patients was shown in Fig. 1. The lenses implanted in the study group were SN60 WF (50 eyes) (Alcon, Fort Worth, TX), iSert Micro251 (10 eyes) (HOYA, Tokyo, Japan) and ZCB00V (4 eyes) (Abbott Medical Optics, Santa Ana, CA).

Table 1 shows preoperative patient data. The mean magnitude of keratometric astigmatism was 0.76 ± 0.46 D (range 0.03 to 2.76 D), and the mean axial length was 23.67 ± 1.69 mm (range 20.98 to 27.55 mm). Astigmatism types were categorized as against-the-rule (ATR) (steepest meridian 0 to 29° or 150 to 180°), with-the-rule (WTR) (steepest meridian 60 to 119°), or oblique (steepest meridian 30 to 59° or 120 to 149°), and the number of eyes for each group was 23, 19, and 22, respectively.

**Table 1:** Preoperative patients’ data

Parameter	Mean ± SD	Range
Age (year)	71.8 ± 9.9	33–92
Sex (male: female)	25: 25	
Right: Left	33: 31	
Keratometric astigmatism (D)	0.76 ± 0.46	0.03–2.76
Type of keratometric astigmatism (eyes)		
ATR (0–29°, 150–180°)	23	
WTR (60–119°)	19	
oblique (30–59°, 120–149°)	22	
Axial length (mm)	23.67 ± 1.69	20.98–27.55

*SD* standard deviation, *D* diopter, *ATR* against-the-rule, *WTR* with-the-rule

**Fig. 2:**
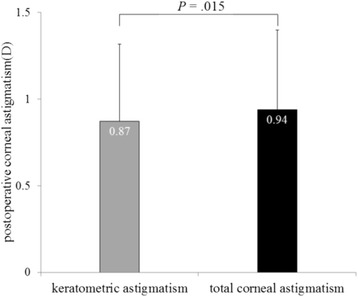
The mean magnitude of postoperative keratometric and total corneal astigmatism was significantly different (*P* = .015, paired *t*-test)

Table 2 shows postoperative patient data. The mean magnitude of objective refractive, keratometric and total corneal astigmatism was 0.92 ± 0.48 D, 0.87 ± 0.44 D, and 0.94 ± 0.46 D, respectively. Total corneal astigmatism was significantly larger than keratometric astigmatism (*P* = .0015, paired *t*-test) (Fig. 2). The mean magnitude of difference was 0.70 ± 0.40 D between refractive and keratometric astigmatism, and 0.63 ± 0.38 D between refractive and total corneal astigmatism, with a significant difference between them (*P* = .019, paired *t*-test).

**Table 2:** Postoperative patients’ data

Parameter	Mean ± SD	Range
Postoperative days (days)	36.0 ± 12.0	20–77
Spherical equivalent refraction (D)	−1.03 ± 1.42	−5.88–1.48
Refractive astigmatism (D)	0.92 ± 0.48	−2.28–−0.12
Keratometric astigmatism (D)	0.87 ± 0.44	0.18–2.46
Total corneal astigmatism (D)	0.94 ± 0.46	0.09–2.68
Difference between refractive and keratometric astigmatism (D) (Mean vector)	0.70 ± 0.40 (0.30 Axis 164°)	0.09–1.72
Difference between refractive and total corneal astigmatism (D) (Mean vector)	0.63 ± 0.38 (0.12 Axis 135°)	0.04–1.81

*SD* standard deviation, *D* diopter

We also calculated the mean difference in magnitude and vector between refractive and each of two types of corneal astigmatism according to the types of preoperative keratometric astigmatism, such as ATR, WTR or oblique astigmatism. The mean difference in magnitude and vector between refractive and keratometric astigmatism were 0.58 ± 0.32 D (0.14 D Axis126°), 0.62 ± 0.31 D (0.44 D Axis2°), 0.89 ± 0.48 D (0.52 D Axis165°), respectively. The vector difference between refractive and total corneal astigmatism were 0.62 ± 0.34 D (0.29 D Axis103°), 0.52 ± 0.28 D (0.22 D Axis5°), 0.75 ± 0.48 D (0.32 D Axis153°), respectively. As for the mean difference in magnitude between postoperative refractive and total corneal astigmatism, there were significant differences in eyes with WTR or oblique astigmatism (*P* = .006, *P* = .007), but not in eyes with ATR astigmatism (*P* = .330).

**Fig. 3:**
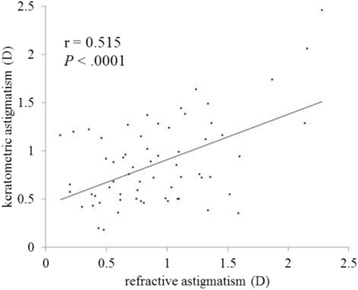
A significant correlation was observed between the magnitude of postoperative refractive and keratometric astigmatism (Pearson’s correlation coefficient; r = 0.515, *P* < .0001)

**Fig. 4:**
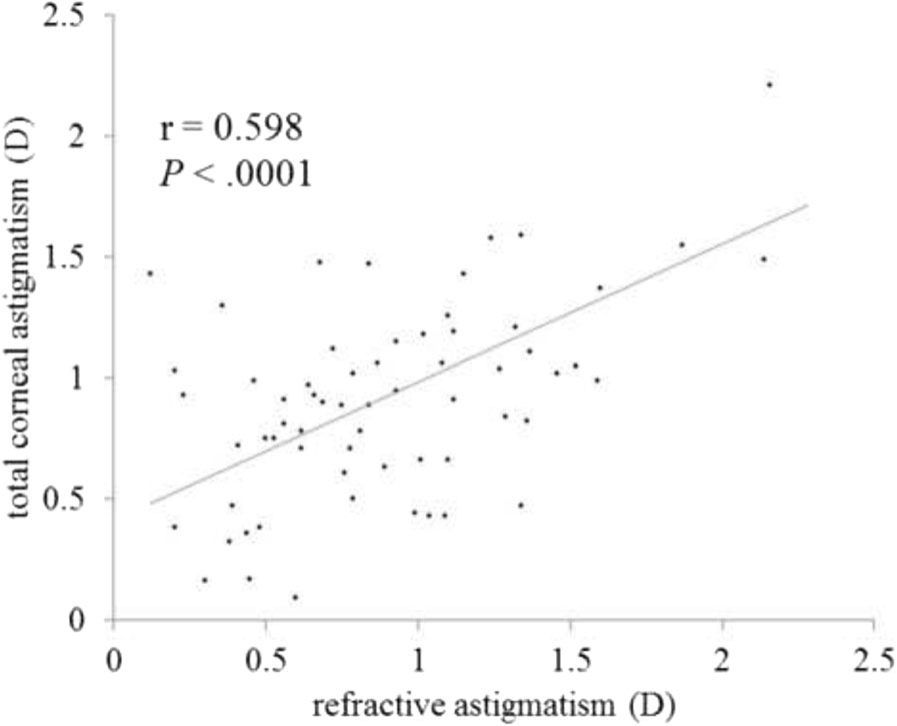
A significant correlation was observed between the magnitude of postoperative refractive and total corneal astigmatism (Pearson’s correlation coefficient; r = 0.598, *P* < .0001)

**Fig. 5:**
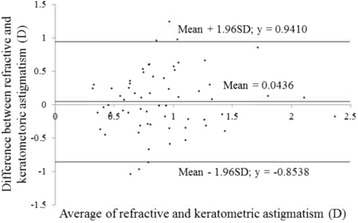
Differences between the magnitude of postoperative refractive and keratometric astigmatism plotted against their average (Bland-Altman plots)

**Fig. 6:**
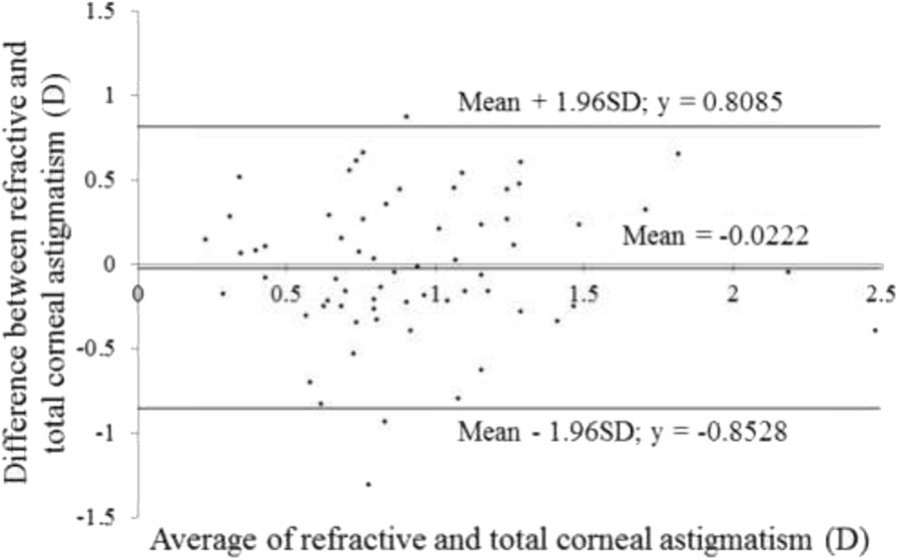
Differences between the magnitude of postoperative refractive and total corneal astigmatism plotted against their average (Bland-Altman plots)

**Fig. 7:**
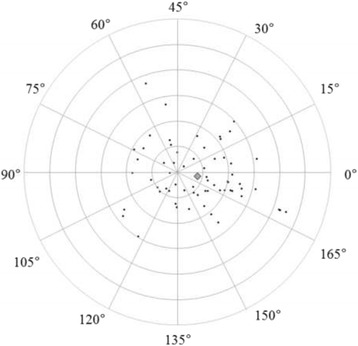
Double-angle plots of postoperative refractive astigmatism. The mean vector of the astigmatism (represented by the grey rhombus, larger than the other plots) was 0.41 D axis 175° (each ring = 0.5 D, outer ring = 3.0 D)

**Fig. 8:**
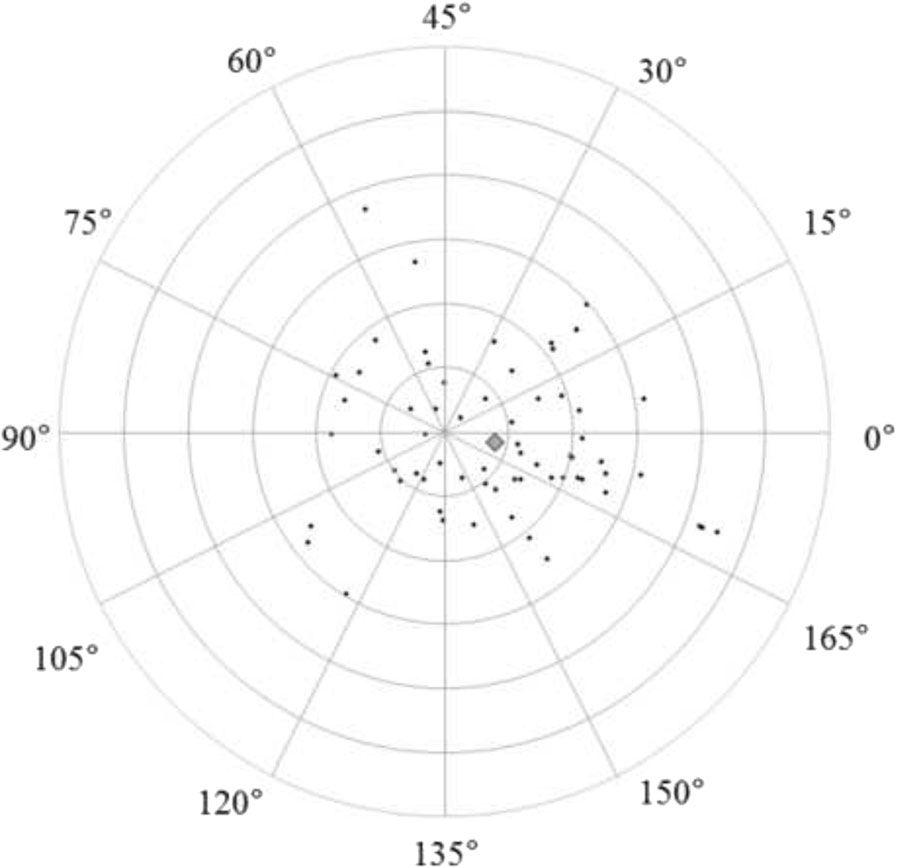
Double-angle plots of postoperative keratometric astigmatism. The mean vector of the astigmatism (represented by the grey rhombus larger than the other plots) was 0.16 D axis 13° (each ring = 0.5 D, outer ring = 3.0 D)

**Fig. 9:**
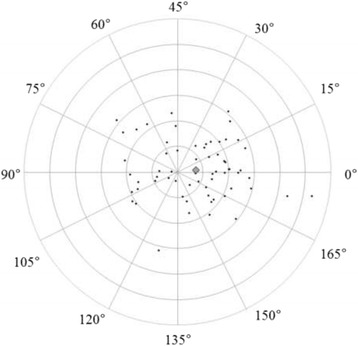
Double-angle plots of postoperative total corneal astigmatism. The mean vector of the astigmatism (represented by the grey rhombus larger than the other plots) was 0.38 D axis 4° (each ring = 0.5 D, outer ring = 3.0 D)

**Fig. 10:**
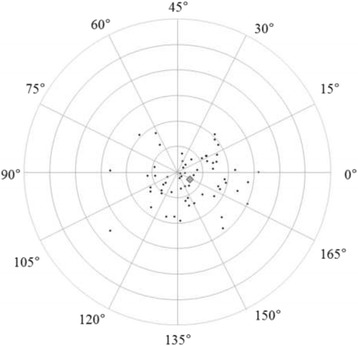
Double-angle plots of difference in vector between postoperative refractive and keratometric astigmatism. The mean vector of the difference (represented by the grey rhombus larger than the other plots) was 0.30 D axis 164° (each ring = 0.5 D, outer ring = 3.0 D)

**Fig. 11:**
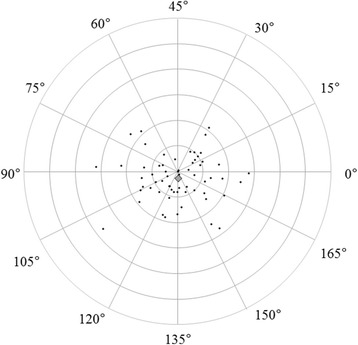
Double-angle plots of difference in vector between postoperative refractive and total corneal astigmatism. The mean vector of the difference (represented by the grey rhombus larger than the other plots) was 0.12 D axis 135° (each ring = 0.5 D, outer ring = 3.0 D)

Figures 3 and 4 show correlations between postoperative refractive and two types of corneal astigmatism (keratometric and total corneal astigmatism). The correlation between refractive and total corneal astigmatism (r = 0.598, *P* < .0001, Fig. 4) seemed stronger than that between refractive and keratometric astigmatism (r = 0.515, *P* < .0001, Fig. 3). Figures 5 to 6 are the Bland-Altman plots showing the relation between postoperative refractive and two types of corneal astigmatism, with the mean values of individual measurements plotted on the horizontal axis and the differences of individual measurements plotted on the vertical axis. It was found that the correlation between refractive and total corneal astigmatism (Fig. 6) is stronger than that between refractive and keratometric astigmatism (Fig. 5). Figures 7 to 9 show doubled-angle plots for each astigmatism. The mean vector of postoperative refractive, keratometric, and total corneal astigmatism was 0.42 D axis 175° (Fig. 7), 0.16 D axis 15° (Fig. 8), and 0.39 D axis 5° (Fig. 9), respectively. Figure 10 shows difference in vector between postoperative refractive and keratometric astigmatism. The mean vector was 0.30 D axis 164°. Figure 11 shows difference in vector between postoperative refractive and total corneal astigmatism. The mean vector was 0.12 D axis 135°. When compared between Figs. 10 and 11, the mean difference in vector between refractive and total corneal astigmatism was closer to 0 D. Table 3 shows the patient data for two groups separated by the magnitude of difference between postoperative refractive and total corneal astigmatism: greater than 0.5 D and less than 0.5 D, respectively. Statistical analysis showed no differences between the two groups in terms of patient age, postoperative spherical equivalent, postoperative refractive, keratometric, and total corneal astigmatism, axial length, or IOL power.

**Table 3:** Patients’ data of two groups

Parameter (postoperative data)	greater than 0.5 D	less than 0.5 D	*P* value
Total number (eyes)	36 (56%)	28 (44%)	-
Age (year)	73.6 ± 10.1	69.4 ± 9.4	.093
Spherical equivalent refraction (D)	−0.80 ± 1.16	−1.33 ± 1.68	.143
Refractive astigmatism (D)	0.92 ± 0.55	0.91 ± 0.39	.868
Keratometric astigmatism (D)	0.93 ± 0.48	0.81 ± 0.38	.296
Total corneal astigmatism (D)	0.97 ± 0.50	0.90 ± 0.40	.553
Axial length (mm)	23.44 ± 1.45	23.97 ± 1.94	.209
IOL power (D)	21.38 ± 3.50	20.09 ± 3.67	.159

*D* diopter

## Discussion

Using AS-OCT, we examined the anterior and posterior corneal curvatures in pseudophakic eyes after phacoemulsification. There are several tools available for the measurement of anterior and posterior corneal curvatures. Among them, OCT-based topography boasts the shortest measurement time as well as high resolution. Tang et al. reported that the repeatability of corneal power measurements obtained using a Fourier-domain OCT system (RTVue, Optovue, Inc. Fremont, CA) was comparable to that of measurements obtained by Placido-ring topography [18]. Szalai et al. reported that AS-OCT had better reliability for measurements of posterior corneal power, anterior and posterior corneal astigmatism, and apical pachymetry in comparison to Scheimpflug imaging [13]. Because the repeatability and reliability of OCT-based topography have been established [13, 18], we used the AS-OCT to examine both anterior and posterior corneal curvatures.

In clinical practice, we sometimes encounter unexpected postoperative refractive astigmatism after cataract surgery even with toric IOLs. When we use toric IOL in cataract surgery, a particular IOL model is selected by assuming that astigmatism is derived entirely from the cornea and crystalline lens. That is to say, if other factors producing astigmatism exist, they must cause unexpected postoperative refractive astigmatism. Analyzing postoperative refractive astigmatism of unknown origin may help more accurate astigmatism correction. In this study, we researched influence of posterior corneal astigmatism on postoperative refractive astigmatism by comparing keratometric and total corneal astigmatism in pseudophakic eyes after non-toric IOL implantation. We found that the mean magnitude of keratometric and total corneal astigmatism were significantly different (Fig. 2). Moreover, the correlation between postoperative refractive and total corneal astigmatism was stronger than that between postoperative refractive and keratometric astigmatism (Figs. 3, 4, 5 and 6). Theoretically, considering that astigmatism is derived entirely from the cornea and crystalline lens, refractive astigmatism must be equal to corneal astigmatism, in cases of non-toric IOL insertion eyes. Therefore, the above findings imply that the relationship between refractive and total corneal astigmatism is more theoretical than that between refractive and keratometric astigmatism. We also calculated the difference between postoperative refractive and two types of corneal astigmatism (keratometric and total corneal astigmatism) (Figs. 10 and 11). Difference between refractive and keratometric astigmatism was also referred to as “internal astigmatism” in some reports [2, 6, 19]. Teus et al. [6] and Tejedor et al. [2] investigated internal astigmatism in eyes implanted with non-toric IOL, and reported that the mean vector of internal astigmatism, corresponding to difference between refractive and keratometric astigmatism in our study, was 0.24 D and 0.38 D, respectively, which coincided approximately with our results (0.30 D).

The mean difference in vector and magnitude between postoperative refractive and total corneal astigmatism was closer to 0 D as compared to that between postoperative refractive and keratometric astigmatism. This suggests that preoperative actual measurement of posterior corneal astigmatism may lead to more accurate postoperative astigmatism correction. When we further examined depending on the types of astigmatism such as ATR, WTR, oblique astigmatism, the mean differences in magnitude and vector between postoperative refractive and total corneal astigmatism was closer to 0 D in eyes with WTR or oblique astigmatism, but not in eyes with ATR astigmatism. We are unaware of the exact reason why eyes with ATR astigmatism had no similar tendency. This issue should be examined in a larger population because the number of eyes with ATR astigmatism was somewhat small in the current study. Although several previous reports have already mentioned the discrepancy in posterior corneal astigmatism between actual and estimated values [4, 5], this is the first report to elucidate the more detailed influences of posterior corneal astigmatism on total refractive error in pseudophakic eyes. Based on the current findings, it can be concluded that incorporating the data of posterior corneal curvature into preoperative IOL power calculation results in better refractive outcomes after cataract surgeries with toric IOLs.

The examination of individual cases showed that 56.0 and 17.2% of eyes showed difference between postoperative refractive and total corneal astigmatism of greater than 0.5 D and 1.0 D, respectively. This means that approximately half of eyes that are planned to undergo toric IOL implantation may exhibit astigmatism correction errors greater than 0.5 D postoperatively even if actual measurement data of posterior corneal astigmatism is incorporated into preoperative IOL power calculation. We tried to find the associated factors which cause the greater difference, but no predictive factors could be identified (Table 3). The difference between postoperative refractive astigmatism and total corneal astigmatism may involve any unknown astigmatism other than corneal and lenticular astigmatism (e.g., retinal, vitreous) [18, 19]. Further studies should be conducted to clarify the causes other than posterior corneal astigmatism which induce postoperative refractive errors.

There are some limitations in this study. First, we only simulated the influence of posterior corneal astigmatism on postoperative refractive astigmatism in pseudophakic eyes after non-toric IOL implantation. Prospective studies should be conducted to compare the surgical outcomes between eyes in which preoperative IOL power calculation is done using total corneal power (including actual measurements of the posterior corneal curvature) and keratometric power (neglecting actual measurement of the posterior corneal curvature). Second, the degree of preoperative corneal astigmatism was relatively small among our study population. Similar research will be necessary also in eyes with larger corneal astigmatism, because candidates for toric IOL implantation generally have considerable corneal astigmatism. Third, we didn’t consider the influence of tilt and dislocation of implanted IOLs on postoperative refraction. There is a possibility that these factors affect postoperative refractive astigmatism. Further studies should also be conducted to elucidate this point.

## Conclusions

This study showed that the relationship between refractive and total corneal astigmatism is more intimate than that between refractive and keratometric astigmatism in pseudophakic eyes, and the vector difference is closer to 0 D in the former than in the latter. If we use actual measurements of the posterior corneal curvature for evaluations prior to cataract surgery, the amount of unexpected postoperative refractive astigmatism might be reduced, resulting in improved uncorrected visual acuity. However, even after eliminating the discrepancy between actual and estimated values of posterior corneal astigmatism, some amount of postoperative refractive astigmatism of unknown origin persisted. Our findings warrant further investigation to find causes of unexpected astigmatism correction errors beyond posterior corneal astigmatism in order to improve visual function after toric IOL implantation.

## Abbreviations

AS-OCT: Anterior segment optical coherence tomography
ATR: Against-the-rule
D: Diopter
IOL: Intraocular lens
SD: Standard deviation
WTR: With-the-rule

## References

[1] Hayashi K, Manabe S, Yoshida M, Hayashi H (2010). Effect of astigmatism on visual acuity in eyes with a diffractive multifocal intraocular lens. J Cataract Refract Surg.

[2] Tejedor J, Guirao A (2013). Agreement between refractive and corneal astigmatism in pseudophakic eyes. Cornea.

[3] Frings A, Katz T, Steinberg J, Druchkiv V, Richard G, Linke SJ (2014). Ocular residual astigmatism: effects of demographic and ocular parameters in myopic laser in situ keratomileusis. J Cataract Refract Surg.

[4] Koch DD, Ali SF, Weikert MP, Shirayama M, Jenkins R, Wang L (2012). Contribution of posterior corneal astigmatism to total corneal astigmatism. J Cataract Refract Surg.

[5] Ho JD, Tsai CY, Liou SW (2009). Accuracy of corneal astigmatism estimation by neglecting the posterior corneal surface measurement. Am J Ophthalmol.

[6] Teus MA, Arruabarrena C, Hernández-Verdejo JL, Sales-Sanz A, Sales-Sanz M (2010). Correlation between keratometric and refractive astigmatism in pseudophakic eyes. J Cataract Refract Surg.

[7] Ueno Y, Hiraoka T, Beheregaray S, Miyazaki M, Ito M, Oshika T (2014). Age-related changes in anterior, posterior, and total corneal astigmatism. J Refract Surg.

[8] Dubbelman M, Sicam VA, Van der Heijde GL (2006). The shape of the anterior and posterior surface of the aging human cornea. Vision Res.

[9] Ho JD, Tsai CY, Tsai RJ, Kuo LL, Tsai IL, Liou SW (2008). Validity of the keratometric index: evaluation by the Pentacam rotating Scheimpflug camera. J Cataract Refract Surg.

[10] Ho JD, Liou SW, Tsai RJ, Tsai CY (2010). Effects of aging on anterior and posterior corneal astigmatism. Cornea.

[11] Ueno Y, Hiraoka T, Miyazaki M, Ito M, Oshika T (2015). Corneal thickness profile and posterior corneal astigmatism in normal corneas. Ophthalmology.

[12] Koch DD, Jenkins RB, Weikert MP, Yeu E, Wang L (2013). Correcting astigmatism with toric intraocular lenses: effect of posterior corneal astigmatism. J Cataract Refract Surg.

[13] Szalai E, Berta A, Hassan Z, Módis L (2012). Reliability and repeatability of swept-source Fourier-domain optical coherence tomography and Scheimpflug imaging in keratoconus. J Cataract Refract Surg.

[14] Nakagawa T, Maeda N, Higashiura R, Hori Y, Inoue T, Nishida K (2011). Corneal topographic analysis in patients with keratoconus using 3-dimensional anterior segment optical coherence tomography. J Cataract Refract Surg.

[15] Yamaguchi T, Ohnuma K, Tomida D, Konomi K, Satake Y, Negishi K, Tsubota K, Shimazaki J (2011). The contribution of the posterior surface to the corneal aberrations in eyes after keratoplasty. Invest Ophthalmol Vis Sci.

[16] Holladay JT, Moran JR, Kezirian GM (2001). Analysis of aggregate surgically induced refractive change, prediction error, and intraocular astigmatism. J Cataract Refract Surg.

[17] Visser N, Berendschot TT, Bauer NJ, Nuijts RM (2012). Vector analysis of corneal and refractive astigmatism changes following toric pseudophakic and toric phakic IOL implantation. Invest Ophthalmol Vis Sci.

[18] Tang M, Chen A, Li Y, Huang D (2010). Corneal power measurement with Fourier-domain optical coherence tomography. J Cataract Refract Surg.

[19] Srivannaboon S (2003). Internal astigmatism and its correlation to corneal and refractive astigmatism. J Med Assoc Thai.

